# Aberrant methylation of NPY, PENK, and WIF1 as a promising marker for blood-based diagnosis of colorectal cancer

**DOI:** 10.1186/1471-2407-13-566

**Published:** 2013-12-01

**Authors:** Jean-Pierre Roperch, Roberto Incitti, Solène Forbin, Floriane Bard, Hicham Mansour, Farida Mesli, Isabelle Baumgaertner, Francesco Brunetti, Iradj Sobhani

**Affiliations:** 1 Profilome SAS, Paris Biotech 24 rue du Faubourg St Jacques, Paris 75014, France; 2 King Abdullah University of Science and Technology (KAUST), Biosciences Core Laboratory, Thuwal 23955-6900, Saudi Arabia; 3 Laboratoire d’Investigation Clinique (LIC), Henri Mondor Hospital & University Paris-Est, Créteil, France; 4 Department of Gastroenterology and Medical Oncology, Henri Mondor Hospital, Créteil, France; 5 Department of Clinical Oncology, Henri Mondor Hospital, Créteil, France; 6 Department of Surgery, Henri Mondor Hospital, Créteil, France

**Keywords:** Colorectal cancer, Circulating DNA methylation, QM-MSP, Epigenetic markers

## Abstract

**Background:**

DNA methylation is a well-known epigenetic mechanism involved in epigenetic gene regulation. Several genes were reported hypermethylated in CRC, althought no gene marker was proven to be individually of sufficient sensitivity or specificity in routine clinical practice. Here, we identified novel epigenetic markers and assessed their combined use for diagnostic accuracy.

**Methods:**

We used methylation arrays on samples from several effluents to characterize methylation profiles in CRC samples and controls, as established by colonoscopy and pathology findings, and selected two differentially methylated candidate epigenetic genes (NPY, PENK). To this gene panel we added WIF, on the basis of being reported in literature as silenced by promoter hypermethylation in several cancers, including CRC. We measured their methylation degrees by quantitative multiplex-methylation specific PCR (QM-MSP) on 15 paired carcinomas and adjacent non-cancerous colorectal tissues and we subsequently performed a clinical validation on two different series of 266 serums, subdivided in 32 CRC, 26 polyps, 47 other cancers and 161 with normal colonoscopy. We assessed the results by receiver operating characteristic curve (ROC), using cumulative methylation index (CMI) as variable threshold.

**Results:**

We obtained CRC detection on tissues with both sensitivity and specificity of 100%. On serum CRC samples, we obtained sensitivity/specificity values of, e.g., 87%/80%, 78%/90% and 59%/95%, and negative predictive value/positive predictive value figures of 97%/47%, 95%/61% and 92%/70%. On serum samples from other cancers we obtained sensitivity/specificity of, e.g, 89%/25%, 43%/80% and 28%/91%.

**Conclusions:**

We showed the potential of NPY, PENK, and WIF1 as combined epigenetic markers for CRC diagnosis, both in tissue and serum and tested their use as serum biomarkers in other cancers. We optimized a QM-MSP for simultaneously quantifying their methylation levels. Our assay can be an effective blood test for patients where CRC risk is present but difficult to assess (e.g. mild symptoms with no CRC family history) and who would therefore not necessarily choose to go for further examination. This panel of markers, if validated, can also be a cost effective screening tool for the detection of asymptomatic cancer patients for colonoscopy.

## Background

Colorectal cancer (CRC) is one of the most frequent malignant diseases worldwide [1,2] yielding high rate mortality [3]. Early diagnosis of CRC is required to increase the survival rates of patients [4]. Currently, endoscopic examination of the colon is the standard for CRC diagnosis. However, this procedure is invasive, unpleasant, carries a number of associated risks of morbidity and mortality and is inaccurate for screening purposes in the average risk populations [5]. Fecal tests (e.g. occult blood test-FOBT and Fecal altered DNA tests) seeking to detect presence of colorectal tumors are available as a pre-colonoscopy test [6-9]. Although FOBTs can significantly reduce mortality due to CRCs [10,11], these tests are flawed by higher rates of false-negatives and false-positives as referred to colonoscopy [12]. In this context, new specific CRC markers for diagnosis of CRC are needed. Over the last decade, aberrant methylation of CpG islands in the promoter and exon 1 regions of tumor suppressor genes is common mechanism in human cancers [13-15] and suggested that measurement of the methylation level can aid diagnosis [16-18].

In the present study, we propose a panel of tumor-specific methylation genes (NPY, PENK, and WIF1) which in combination show a potential as epigenetic markers for the colorectal cancer diagnosis. We have developed a quantitative multiplex-methylation specific PCR (QM-MSP) to quantitate cumulative methylation of these markers in tissue and serum samples. On serum sample, we suggest that our QM-MSP can help in preselecting the patients having mild symptoms or without CRC family history for colonoscopy and potentially, if validated, for the screening of colorectal cancer.

## Methods

### Human samples

Human samples were collected from individuals referred to the gastrointestinal endoscopy units of several academic hospitals (Table 1). Patients gave informed consent (registered under 04–2004 and revised as CPP-IDF IX-11-019 by CPP, consultative ethical committee in the Ile de France-Est medical district), blood samples were collected prior to colonoscopy. Endoscopy and pathology reports were recorded on anonymized files. Tumor biopsies were obtained under colonoscopy procedures or by using surgical resections. Tissue samples have been frozen at −80°C until DNA was extracted. For each individual, samples were also paraffin-embedded and conserved for pathology analyses. In all cases, samples of normal homologous colonic tissues were similarly conserved. They were used for microsatellite instability analysis and the KRAS mutations which are routinely performed in our hospital before undergoing methylation testing and tumor staging was determined according to the TNM-classification (Additional file 1: Table S1).

**Table 1:** Patients characteristics of clinical studies

	**Characteristic**	**Patients**	**Controls**
Tissue		*n* = 15^*^	
	Sex		
	Male	12	
	Female	3	
	Age (yr)		
	Mean ± SD	70.4 ± 13.5	
	Range	48-93	
	Stage		
	I/II	6 (1/5)	
	III/IV	9 (5/4)	
Serum		*n* = 32	*n* = 161
	Sex		
	Male	21	86
	Female	11	75
	Age		
	Mean ± SD	67.9 ± 12.9	59.1 ± 16.5
	Range	36-94	18-94
	Stage		
	I/II	6 (2/4)	Na
	III/IV	26 (7/19)	Na

*In total 30 tisue samples corresponding to 15 CRC and 15 normal homologous tissues from 15 CRC patients were analyzed.

Na: not applicable.

### Description of the clinical study

**Figure 1:**
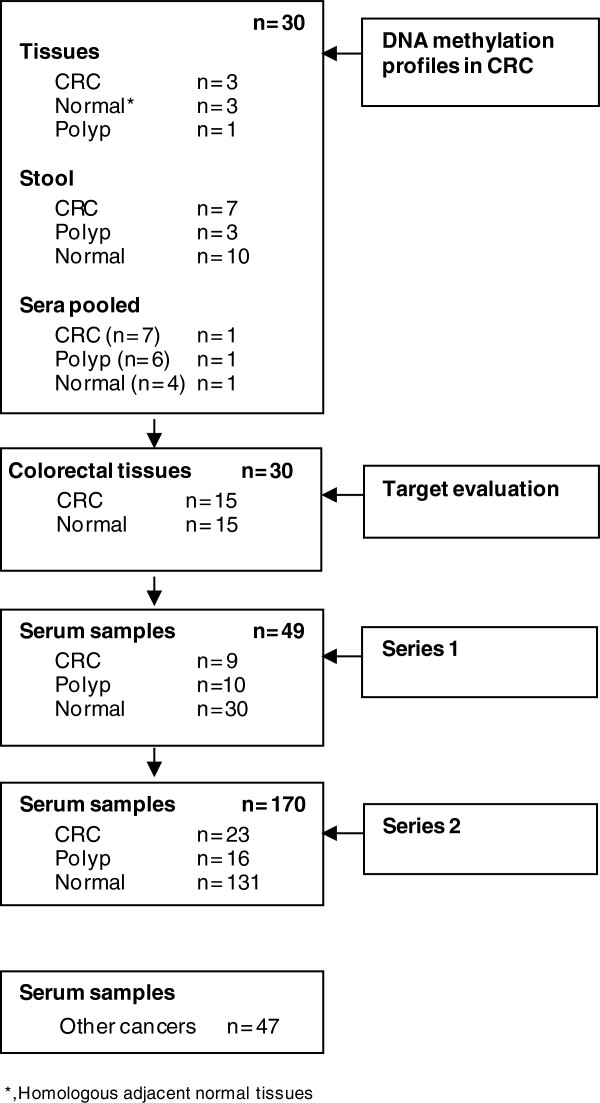
**Schematic representation of the study design.** First target genes have been identified by using Illumina microarray analyses on tissues, stools, sera. Then a multiplex methylated test has been constructed and evaluated on tissue samples. Finally, two validations in sera have been then performed (Series 1 and 2).

First, a comprehensive DNA methylation profiling was performed on DNA from 30 tissues, stools and serum samples using Illumina goldengate methylation arrays that contain 1,505 markers (CpG loci) within 807 cancer-related genes (Illumina, CA). We selected NPY, PENK on the basis of their hypermethylation and their power to discriminative normal from CRC patients. Secondly, these candidate genes, together with WIF1gene that we selected based on evidence from literature [19-31], were evaluated in a multiplex assay on an additional 15 normal/cancer paired colonic tissues. Thirdly, validations of the multiplex assay were carried out on the two independent series of sera (Table 2). Series 1 contained 49 serum samples including 9 patients with CRC, 10 patients with large polyp adenomatous (1 cm in diameter or more) at colonoscopy with 30 individuals with normal colonoscopy. Series 2 validation was carried out on 170 serum samples from 23 patients presenting with CRC, 16 patients with large polyp adenomatous, and 131 control individuals with tumor-free at colonoscopy (Figure 1). In the Series 3, we assayed 47 patients suffering from a digestive or extra digestive tumor other than CRC such as breast, prostate, kidney, bladder, liver, esophagus, pancreas, cholangiocarcinoma and stomach cancers.

**Table 2:** Clinicopathologic characteristics in serum samples of patients with CRC and healthy control

	**Characteristic**	**CRC**	**Polyps**	**Controls**
Series 1		*n = 9*	*n = 10*	*n = 30*
	Sex			
	Male	3	6	15
	Female	6	4	15
	Age			
	Mean ± SD	66.1 ± 17.2	71.5 ± 11.8	62. 1 ± 17.3
	Range	36-94	56-91	22-91
	Stage			
	I/II	4 (1/3)	Na	Na
	III/IV	5 (1/4)	Na	Na
Series 2		*n = 23*	*n = 16*	*n = 131*
	Sex			
	Male	18	10	71
	Female	5	6	60
	Age			
	Mean ± SD	68.6 ± 11.2	61.8 ± 9.2	58.5 ± 16.3
	Range	51-84	52-85	18-94
	Stage			
	I/II	2 (1/1)	Na	Na
	III/IV	21 (6/15)	Na	Na
Series 1 + 2 pooled		*n = 32*	*n =26*	*n = 161*
	Sex			
	Male	21	16	86
	Female	11	10	75
	Age			
	Mean ± SD	67.9 ± 12.9	65.5 ± 11.1	59.1 ± 16.5
	Range	36-94	52-91	18-94
	Stage			
	I/II	6 (2/4)	Na	Na
	III/IV	26 (7/19)	Na	Na

Na: not applicable.

### DNA isolation and bisulfite modification

DNA was isolated from colonic tissues and stool samples by using a QIAamp DNA Mini Kit (Qiagen), and a QiAamp DNA stool mini kit (Qiagen), respectively. DNAs were isolated by using a ZR Serum DNA kit (Zymo Research) according to the manufacturer’s protocol and were stored at −20°C until methylation quantification after concentrations were performed using the Eppendorf BioPhotometer. Bisulfite treatment was adopted to transform unmethylated cytosine nucleotides into thymidine without changing methylated cytosines. This was carried out after DNA was chemically modified with sodium bisulfite at 50°C in the dark for 16 hours by using an EZ DNA Methylation kit (Zymo Research).

### Quantitative methylation-specific PCR amplification

Modified DNA was analyzed by QS-MSP (quantitative singleplex-methylation specific PCR), and the QM-MSP (quantitative multiplex-methylation specific PCR). All PCR reactions were performed using an ABI prism 7900 HT sequence detector (Applied Biosystems). For each PCR run, a master-mix was prepared, primers and probes for WIF1, NPY and PENK have been designed, and a primer/probe set of albumin not containing CpG sites was used for normalizing the DNA amounts (Additional file 2: Table S2). The thermal cycling conditions included an initial denaturizing step at 95°C 48 cycles for 15 s and at 60°C for 1 min. Bisulfite methylated DNA (Zymo Research) was used as calibrator and positive control. DNA free distilled water was used as negative control. The relative level of methylation was determined by the 2^-∆∆Ct^ method as described in supplementary data and the efficiency of reactions was determined by plotting in logarithmic scale the amounts of methylated DNA versus the corresponding Cts (cycle threshold) as baseline curves of the genes.

### Bisulfite genomic sequencing

The PCR products of albumin, NPY, PENK, and WIF1 genes were purified before submission to the sequencing process of both strands by using BigDye Terminator Cycle Sequencing kit (Applied Biosystems) according to the manufacturer’s instructions. The sequence reactions were run and analyzed on an ABI 3100 Genetic Analyzer (Applied Biosystems).

### DNA methylation profiling using Illumina Goldengate methylation bead arrays

500 ng of bisulfite-converted DNA were probed on the Illumina Goldengate Methylation Cancer Panel I. A total of 30 DNA samples were assayed on the Illumina platform. Totally, there were seven tissue samples (3 colon cancer tissue, 1 large polyp tissue and 3 paired adjacent normal tissues), 20 stools samples (7 CRC patients, 3 individuals with large polyp adenomatous and 10 individuals with normal colonoscopy), and three pools of their serum DNA samples including colon cancer patients, patients with polyp adenomatous and individuals with normal colonoscopy. The values for each CpG site as a value in the range of 0 –100.0% of methylation after subtracting background of negative controls on the array and taking the ratio of the methylated signal intensity to the sum of both methylated and unmethylated signals were provided by Illumina together with a technical p-value.

### Data analysis

1) Selection of biomarker candidates on the microarray data: we first flagged the features on the array that did not pass the quality score recommended by the manufacturer; the number of non-flagged was higher in tissues (1300 to 1400) than in serum (1200 to 1400) or stools (1000 to 1200). Hierarchical clustering analysis revealed a striking difference in methylation between specimens taken from normal colonoscopy individuals and those from cancer patients, for both tissues and effluent samples. To investigate the results at the simple locus level, we proceeded as follows: we computed the averages of each locus’ methylation values across all samples for tissue and stool in each category of normal (N) and cancer (Ca) individuals; for blood, we retained the value provided by Illumina for the single pooled sample assayed. Differences (Ca-N) between cancer and normal tissues or milieus were computed and the results were ranked according to Ca-N. Then for each of tissue, serum and stool we selected the most differentially methylated loci by taking the top decile in the Ca-N ranked differences. We performed cross comparisons between the three lists so obtained by intersecting those lists. We found 5 CpG loci in the three-wise intersection, above the number expected (p_val 0.019).

2) Performance for CRC discrimination of combined NPY/PENK/WIF1: we computed a cumulative methylation index (CMI) consisting in the sum of the three methylation values for each sample and used it as a varying threshold for constructing a ROC curve. Specificity is calculated as the number of true negatives divided by the number of true negatives plus false positives. Sensitivity is calculated as the number of the true positives divided by the number of true positives plus false negatives. NPV is calculated as the number of the true negatives divides by the number of true negatives plus false negatives. PPV is calculated as the number of the true positives divided by the number of true positives plus false positives.

## Results

### Selection of candidate biomarkers by DNA methylation-array

To screen for candidate biomarkers, we carried out a microarray study on tissue, serum and stool samples. We found 5 CpG loci, distributed among 4 genes in the intersection of the most differentially methylated loci; we selected PENK and NPY in that gene set (Additional file 3: Figure S1). We brought those two genes, together with WIF, into a QM-MSP assay for evaluation and clinical validation.

### Verification of DNA promoter methylation status by bisulfite sequencing

Both methylated and unmethylated alleles were identified and fully characterized in a series of 12 PCR products through a bi-directional sequencing process and specific forward and reverse primers that did not contain CpG sites (Additional file 2: Table S2). As illustrated for WIF1 marker, sequencing results revealed that all CpG covering the amplicon in tumor samples were uniformly methylated. By contrast, in adjacent normal tissues all CpG were uniformly unmethylated showing the presence of thymidine nucleotides instead of cytosine on CpG sites, which suggests that bisulfite induced conversion (Additional file 4: Figure S2).

### Efficiency and specificity of the real-time QM-MSP assay

**Figure 2:**
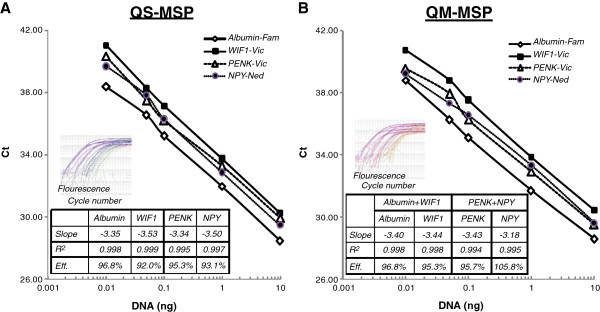
**Efficiency of quantitative simplex (QS) and multiplex (QM) methylation-specific PCR.** The diagrams illustrate comparison of both methods, namely **A**: quantitative multiplex methylation-specific PCR (QM-MSP) and **B**: quantitative singleplex methylation-specific PCR (QS-MSP). The reactions have been performed in duplicate. We used a mixture of primers and hydrolysis methylated probe specific to only amplify methylated alleles of Albumin, WIF1, PENK, and NPY genes along with a titration of human genomic DNA at various concentrations ranging from 10 up to 0.01 ng/well. On each dilution, the cycle threshold (Ct) was determined for standard DNA. Nearly identical Ct values for each DNA dilution indicate uniform primer performance over 3 logs. The slope of −3.32 (100% efficiency) reflects a 2-fold amplification of DNA per cycle corresponding to a high efficiency. The correlation coefficient R^2^ of 0.99 shows a high degree of linearity over the entire range.

We evaluated the performance of two different PCR-based assays, quantitative singleplex-MSP (QS-MSP) and quantitative multiplex-MSP (QM-MSP), in order to quantify the methylation levels of NPY, PENK, and WIF1. For co-amplifying two methylation-specific DNA targets in real-time, we used the associations of Fam/Vic and Ned/Vic fluorophore probes as each probe presents a strong individual spectral intensities with limited overlapping absorption spectra. We compared QS-MSP and QM-MSP to determine which assay agreed best with the detection thresholds (Ct) on a serial dilution experiment from 10 ng to 10 pg of methylated DNA. Both QS-MSP and QM-MSP gave similar cycle threshold (Ct) values for each dilution point (data not shown) with similar high amplification efficiency (Figure 2A, 2B).

### QM-MSP assays in paired normal and tumor tissues

**Figure 3:**
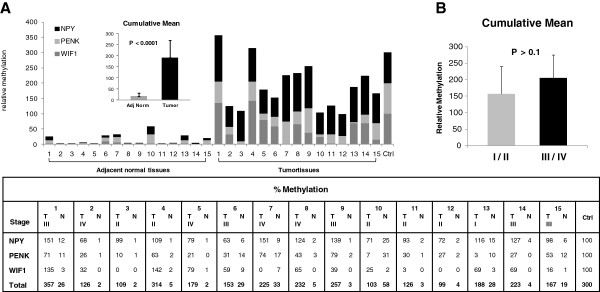
**Cumulative promoter hypermethylation of WIF1, PENK, and NPY in adjacent normal and colorectal carcinoma tissues. A**, cumulative methylation levels in tumor tissues and homologous adjacent normal tissues in an independent experiment, paired samples (n = 15) of adjacent normal and tumor tissue were quantified by using QM-MSP. Samples were scored for cumulative methylation index (CMI) of three genes. Results are plotted as stacked bar graphs. Differences of CMI between homologous adjacent normal tissues (Norm) versus tumor tissues (Tumor) were significant (P < 0.0001, Student-test). Plotted is the mean (± SD; bars) amount of CMI in Norm (mean = 15.07 ± 16.60) and in Tumor tissues (mean = 190.57 ± 77.65). Actual percentage of methylation values are listed in the table for tumor (T), adjacent normal (N), and control (Ctrl). **B**, Cumulative methylation of target genes by QM-MSP of DNA tissue samples from CRC patients. Mean cumulative methylation in stage I / II and in stage III / IV are shown. Differences between tumor stages were not significant (P > 0.09, Student-test). Plotted is the mean (± SD; bars) amount of cumulative methylation in Stages I / II (mean = 156.74 ± 83.96) versus Stages III / IV (mean = 213.14 ± 68.66).

We used two multiplex assays, namely Alb-Fam/WIF1-Vic and the NPY-Ned/PENK-Vic, to measure methylation of our three biomarkers in a set of 15 paired normal and tumor tissue samples. We set thresholds for the levels of methylation of, respectively, 25% for NPY, 17% for PENK, and 7% for WIF1 and obtained the following corresponding performances: NPY displayed 100% sensitivity (Se) and 100% specificity (Sp), PENK displayed 80%/93.3%, and WIF1 displayed 73.3%/93.3%, respectively (Additional file 5: Table S3). The sum of all methylation values across the three genes or cumulative methylation index (CMI), ranged between 2% and 58% in adjacent normal tissues and was greater or equal to 99% in carcinoma tissues (Figure 3A). The mean values (±SD) of CMI in adjacent normal tissues (15.07 ± 16.60) were significantly lower than those in carcinoma tissues (190.57 ± 77.65; p < 0.0001, Student-test; Figure 3). With a CMI threshold of 58%, a Se of 100% (15 of 15) and a Sp of 100% (15 of 15) were obtained (Additional file 5: Table S3) and no significant differences of CMI related to the stages of carcinoma could be observed according to TNM staging: 156.74 ± 83.96 for stages I/II and 213.14 ± 68.66 for stages III/IV (*P* = 0.09, Student-test; Figure 3B).

### Validation of QM-MSP test in the sera for the detection of CRC

We measured NPY, PENK and WIF1 by QM-MSP on two hundred and sixty six serum samples and assayed the discrimination power of their CMI. The set of samples consisted in a preliminary clinical set (Series 1) that included 49 individuals (30 presenting with normal colonoscopy, 10 with large adenomatous polyps and 9 with CRC) and in a second clinical set (Series 2) including 170 individuals (131 presenting with normal colonoscopy, 23 with CRC, 16 with large polyp adenomatous) (Table 2).

**Figure 4:**
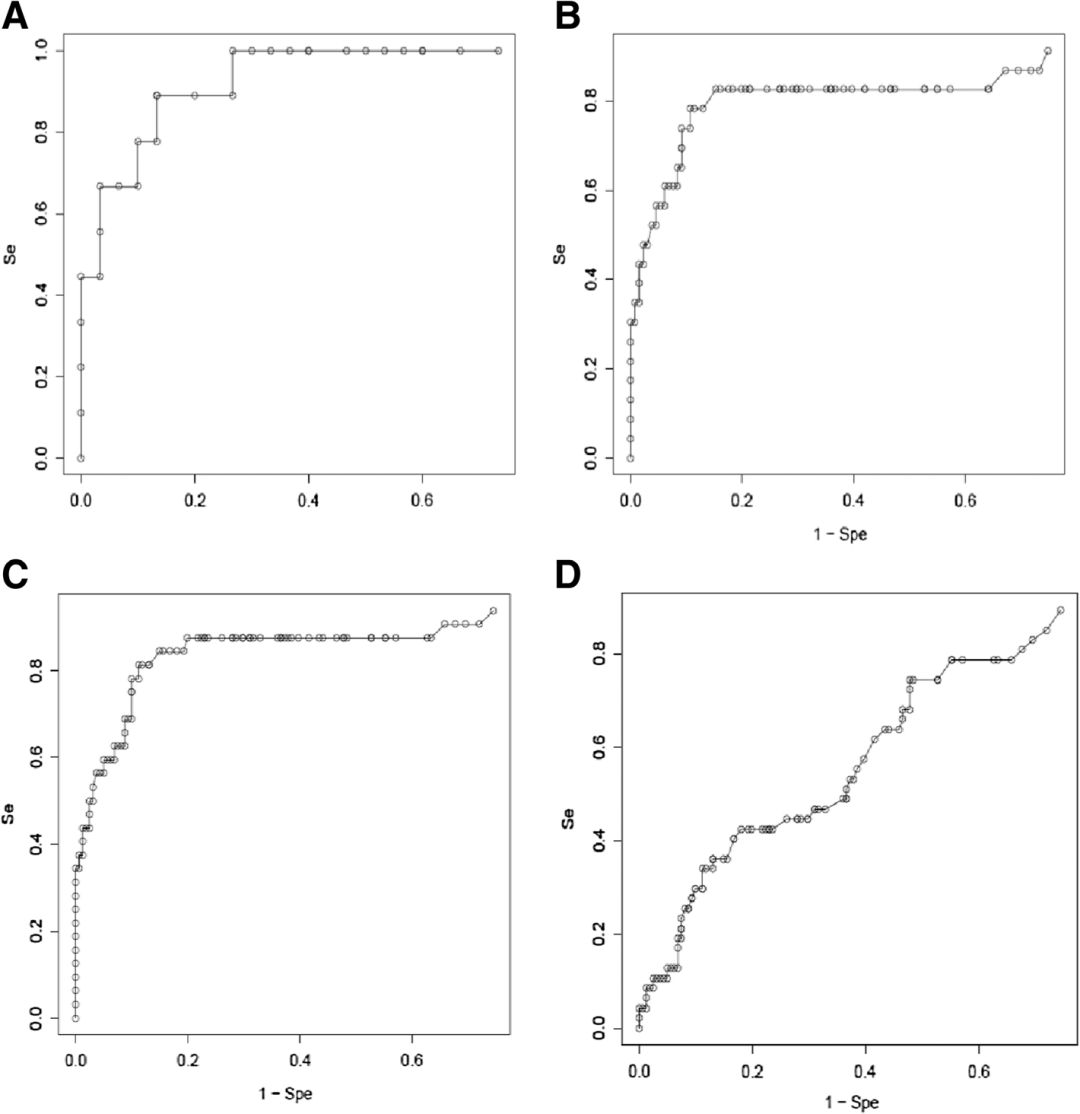
**ROC curve relative to the cumulative methylation index.** Sum of three (WIF1, NPY, PENK) methylation indexes are used to establish ROC curves corresponding to Series 1 **(A)**, Series 2 **(B)**, total of both series **(C)** for CRC and Series 3 **(D)** for other cancers.

CMI values were used for calculating the Specificity (Sp) versus the Sensitivity (Se) depending on various thresholds and the ROC (Receiver Operating Characteristic) diagrams were constructed. For each of the two series, we obtained similar ROC profiles for CRC detection (Figure 4A, 4B). To highlight key trade-offs between Se and Sp, we consider CMI thresholds for having high Se (e.g. Se about 90%) and high Sp (e.g. Sp about 90% or Sp about 95%). So, pooling the two series (Figure 4C), we obtain sensitivity/specificity figures of, respectively, 87%/80%, 78%/90% and 59%/95% (Table 3), and NPV/PPV figures of 97%/47%, 95%/61% and 92%/70% (as computed without factoring the prevalence, since the population is already symptomatic). No significant relationship could be identified between serum CMI rates and TNM staging (Additional file 6: Figure S3).

**Table 3:** Cumulative promoter hypermethylation of WIF1, PENK, and NPY DNA in serum

	**CMI**	**Specificity**	**Sensitivity**
Series 1	0.62	73 (22/30)	100 (9/9)
	0.68-0.75	>80	>80
	1.15	90	66
	2.85	95	66
Series 2	0.01	25 (33/131)	91 (21/23)
	0.60-0.73	>80	>80
	0.94	90	75
	2.48	96	52
Pooled 1 + 2	0.05	34 (55/161)	94 (30/32)
	0.62-0.85	>80	>80
	0.94	90	75
	2.01	95	59

Sensitivity (Se) and Specificity (Sp) are shown at several CMI thresholds for both CRC series: Se > 90% (CMI = 0.62 for Series 1 and 0.01 for Series 2), Sp > 90% (1.15 and 0.94) and Sp > 95% (2.85 and 2.48). We note the existence of two similar numerical ranges for the thresholds (0.68-0.75 for Series 1 and 0.60-0.73 for Series 2) where both Sp and Se are high (>80%). CMI values are the cumulative methylation index of three genes.

### QM-MSP test in the sera for testing other cancers

To assess the specific relevance of our gene panel to CRC we assayed in the same way forty seven serum samples from patients with cancers other than CRC obtaining sensitivity/specificity values of, e.g., 89%/25%, 43%/80% and 28%/91% (Figure 3D).

## Discussion

Here, we have shown that methylation profiling based on beadchip arrays is an effective method for selecting the genes with promoter methylation (i.e. NPY and PENK). Using our QM-MSP, we found a significant difference in the methylation levels of NPY, PENK, and WIF1 between CRC and normal tissue and sera. On serum, the test performs CRC detection with sensitivity/specificity values of 87%/80% (higher sensitivity) or 78%/90%, and 59%/95% (higher specificity).

Epigenetic abnormalities leading to gene silencing, are a common occurrence in many malignancies [32]. They can be considered as a way to modulate gene activity, alternative or complementary way to gene mutations. The Wnt signaling pathway is critical for the regulation of colonic crypt renewal and homeostasis [33].The deregulation of crypt homeostasis, together with the loss of APC function by mutations, is known to initiate colorectal carcinogenesis [34,35]. In the epigenetic field, a large number of studies have suggested that promoter methylation-induced silencing of Wnt pathway antagonist genes constitute an “epigenetic gatekeeper”, leading to constitutive Wnt signaling in many cancers [36] and colorectal cancer [37,38] with many CpG islands reportedly affected in both tumors and in pre-cancerous lesions [39].

We have focused on the Wnt antagonist gene WIF1 (Wnt inhibitor factor 1) because it has been reported that the epigenetic silencing of this gene induces an aberrant activation of the Wnt signaling pathway in many cancers. This gene encodes a secreted Wnt antagonist sequestering secreted Wnt proteins and inhibits their activities, limiting carcinogenesis in human [19,20]. Loss of WIF1 expression leads to aberrantly activate Wnt signaling, which is associated with cancer and could act as a tumor suppressor gene [21,22]. WIF1 expression was found to be frequently down-regulated in hepatocellular carcinoma; this down-regulation could be attributed to hypermethylation of the WIF1 promoter [23]. In osteosarcomas, silencing of WIF1 by promoter hypermethylation was associated with loss of differentiation and increased proliferation [24]. Recent studies demonstrate that the WIF1 gene is down-regulated or silenced in astrocytomas [25], the most common tumors of the central nervous system, and in cervical cancer [26], both by aberrant promoter methylation. WIF1 was reported as frequent target of epigenetic inactivation in several tumors such as lung, prostate, breast, bladder cancers [27-29]. In glioblastomas, WIF1 silencing is mediated by genomic deletion, promoter methylation, or both [30]. The WIF1 gene promoter hypermethylation has been reported in circulating DNA isolated from plasma of colorectal adenoma and CRC patients [31].

We presumed that WIF1 could be considered as a target for epigenetic silencing in CRC. Our results from tissues and effluents were consistent with this hypothesis. However, WIF1 alone could not be considered as a unique marker for cancer detection, from effluents, although its discriminative value in tissues was very high. This is the reason why we investigated a larger panel including various other genes. Accordingly, we used Illumina methylated microarray as a genome-wide screening tool for finding hypermethylated genes in CRC and normal colonoscopy patients’ effluents and characterized a panel of less than ten genes including NPY and PENK, which are known to be involved in gastrointestinal tract functions particularly in nutriment uptake and absorptions.

Neuropeptide Y (NPY), a neurotransmitter, acts on the central nervous system as a potent appetite stimulator controlled by the feedback action of both leptin from adipose tissue and ghrelin from the stomach [40,41]. These two peptides are involved in obesity and metabolic syndrome, two conditions clearly increasing the risk of cancers particularly the colon cancer [42]. NPY is involved in cell motion and cell proliferation as well as neuropeptide hormone activity [43]. NPY can reduce the invasive potential of colon cancer cells in vitro [44]. In prostate cancer, the decrease of NPY expression is associated with aggressive clinical behavior [45]. In other studies, NPY was shown to be frequently hypermethylated in neuroblastomas [46], hepatocellular carcinoma tissues [47] and their promoter hypermethylation was correlated with inactivation of gene expression. More recently, DeMorrow and colleagues have demonstrated that the treatment of cholangiocarcinoma cells with NPY as well as in vitro and in vivo decreases both proliferation and migration [48]. The present study reports the evidence of NPY gene involvement in CRC. Although further investigations are required to understand whether hypermethylation is a cause or a consequence of carcinogenesis, it is suggested here to use hypermethylated gene as a blood-based marker.

Proenkephalin (PENK), was originally shown to be expressed in the mature nervous and neuroendocrine systems through opioid pathway, in the regulation of cell death and survival [49]. PENK protein has been shown to act as apoptotic activator particularly under chemotherapy drugs in colon cancer [50,51]. Its expression being down-regulated by Fos and Jun, two proto-oncogenes [52]. PENK was reported to be down-regulated in prostate cancer [53]. PENK is frequently methylated in bladder [54], and pancreatic cancer [55-57]. Although, no study has so far established a direct link between the PENK promoter hypermethylation and the development of CRC, our findings suggest that this gene is frequently hypermethylated in CRC patients’ effluents and might be a valuable biomarker for its detection.

Main advantages of our QM-MSP are an analysis of several gene performed in a single process and a quantification of methylation allowing optimal balancing between sensitivity and specificity. Our clinical study shows that the variation of methylation threshold could offer of tests for diagnosis as well as surveillance of recurrences of CRC. For example, a CMI threshold of 0.05 appears to be more appropriate for diagnosis/monitoring purposes, yielding high sensitivity, detecting the real cancers; a CMI of 2 sets our selection in the higher range of specificity, so limiting the number of unnecessary colonoscopies. We also showed relevance of our gene panel for detecting non colon cancers in a series of 47 patients’ samples, where we obtained sensitivity/specificity of, e.g., 89%/25%, 43%/80% and 28%/91%. However, a limitation of the proposed test is the low rate of adenomatous detection, making it necessary to establish the optimal periodicity for performing the test.

## Conclusions

In this paper we show data indicating that combining the methylation values of NPY, PENK, and WIF1 is potentially useful as a sensitive and specific blood test for identifying among individuals with digestive symptoms, those individuals for whom colonoscopy is recommended. This test, if validated, could be proposed as a cost effective non invasive screening tool for the selection of asymptomatic cancer patients for colonoscopy. The results for other cancers suggest a possible second use for the test for patients who would be positives to the test and negative to colonoscopy, indicating that might undergo other cancer-specific examinations.

## Competing interests

Authors do not have any competing interests for writing this article. JP Roperch is an employee, and I. Sobhani is the main scientific consultant of Company Profilome SAS, Paris.

## Authors’ contributions

Participated in research design: All authors. Conducted experiments: JPR, RI, SF, FLB, and IS. Performed data analysis: JPR and RI. Wrote or Contributed to the writing of the manuscript: JPR, RI, and IS. All authors read and approved the final manuscript to be published.

## Pre-publication history

The pre-publication history for this paper can be accessed here:

http://www.biomedcentral.com/1471-2407/13/566/prepub

## Supplementary Material

Additional file 1: Table S1 Full clinical characteristics in tumor tissue samples and detection K-ras mutations. Mutation screening of the exon 1 of the K-ras gene containing hot spot codons 12 and 13 was assessed from paraffin-embedded tissue blocks of 15 patients diagnosed with colon adenocarcinoma. A short fragment of 80 bp of KRAS gene overlapping the codon 12 and 13 was amplified and then sequenced using the following primer pair: forward, 5’-AGGCCTGCTGAAAATGACTGAATAT-3’ and reverse, 5’-GCTGTATCGTCAAGGCACTCTT-3’. PCR was performed in a reaction volume of 20 μL consisting of 2 μL of 10 ng/μL of DNA sample, 10 μL of 2 X SyberGreen PCR Master Mix (Applied Biosystems), 0.80 μl of 10 μM of forward and reverse primers (400 nmol/L in final concentration) and 6.4 μL of sterile water. Amplifications were performed in duplicate in 96-well plates in a real-time 7900 HT (Applied Biosystems) with as a first step a denaturating at 95 °C for 15 min, then 15 sec at 95 °C, 1 min at 60 °C for 48 cycles. Products were purified and then sequenced in both directions (forward and reverse) using BigDye Terminator Cycle Sequencing kit (Applied Biosystems) according to the manufacturer’s instructions. The primers used for the sequencing were identical to those used for the PCR. The sequence reactions were run and analyzed on an ABI 3100 Genetic Analyzer (Applied Biosystems). Click here for file

Additional file 2: Table S2 Oligonucleotides. Click here for file

Additional file 3: Figure S1 Selection of candidate biomarkers by DNA methylation-array. Left: Venn diagram. Urine, Serum and Tissue lists obtained by taking the top decile in the ranked Ca-N lists. Right: Loci Illumina goldengate IDs. Click here for file

Additional file 4: Figure S2 The bisulfite sequence of the WIF1 promoter. Representative bisulfite sequencing electrophoregram of the WIF1 promoter verifies methylation status assessed by QS-MSP from carcinoma colorectal (Tumor) and adjacent normal tissues (Adj Norm). The diagram above illustrates one of the 15 samples of tumor tissues; cytosine nucleotides underlined in black remain unchanged indicating all sites are methylated in the amplicon product. By contrast, in the homologous normal tissue, only thymidine nucleotides underlined in red are detected instead of cytosine residues due to bisulfite modified DNA which is indicative of unmethylated amplicon products. It is interesting to note that comparison of two sequences of normal and tumoral tissues indicates that all cytosine at non-CpG sites are converted to thymine resulting entirely from DNA modification. This follows after sodium bisulfite treatment when referring to the wild-type WIF1 gene sequence. Click here for file

Additional file 5: Table S3 Gene promoter analysis in adjacent normal and tumor tissues. Optimal threshold values obtained for simple gene and in combination of 3 genes. Taking into account the different degrees of methylation, we set threshold of CMI at 58% to obtain the highest performance in terms of sensitivity and specificity. Click here for file

Additional file 6: Figure S3 Methylation correlated in stages of CRC. Mean cumulative methylation in I / II and III / IV stages of CRC serum samples. Differences between both stages were not significant (P > 0.1, Student-test). Plotted is the mean (± SD; bars) amount of cumulative methylation in I / II stages with mean = 44.40 ± 78.53, versus in III / IV stages with mean = 33.55 ± 61.71. Click here for file
