# Supplementary material for: Aberrant methylation of NPY, PENK, and WIF1 as a promising marker for blood-based diagnosis of colorectal cancer
Source: BMC Cancer. 2013 Dec 1;13:566. doi: 10.1186/1471-2407-13-566 (PMC4219483; doi:10.1186/1471-2407-13-566)
Supplement: Additional file 1: Table S1 — Full clinical characteristics in tumor tissue samples and detection K-ras mutations. Mutation screening of the exon 1 of the K-ras gene containing hot spot codons 12 and 13 was assessed from paraffin-embedded tissue blocks of 15 patients diagnosed with colon adenocarcinoma. A short fragment of 80 bp of KRAS gene overlapping the codon 12 and 13 was amplified and then sequenced using the following primer pair: forward, 5’-AGGCCTGCTGAAAATGACTGAATAT-3’ and reverse, 5’-GCTGTATCGTCAAGGCACTCTT-3’. PCR was performed in a reaction volume of 20 μL consisting of 2 μL of 10 ng/μL of DNA sample, 10 μL of 2 X SyberGreen PCR Master Mix (Applied Biosystems), 0.80 μl of 10 μM of forward and reverse primers (400 nmol/L in final concentration) and 6.4 μL of sterile water. Amplifications were performed in duplicate in 96-well plates in a real-time 7900 HT (Applied Biosystems) with as a first step a denaturating at 95 °C for 15 min, then 15 sec at 95 °C, 1 min at 60 °C for 48 cycles. Products were purified and then sequenced in both directions (forward and reverse) using BigDye Terminator Cycle Sequencing kit (Applied Biosystems) according to the manufacturer’s instructions. The primers used for the sequencing were identical to those used for the PCR. The sequence reactions were run and analyzed on an ABI 3100 Genetic Analyzer (Applied Biosystems). [file 1471-2407-13-566-S1.doc]

| Tissue (n= 15) | Sex | TNM | Stage | Tumor cells, % | K-ras mutations | MSI status |
| --- | --- | --- | --- | --- | --- | --- |
| 1 T443318 | M | T4N1M0 | III | 90 | Wild type (WT) | MSS |
| 2 T443381 | M | T4N1M1 | IV | 50 | WT | MSS |
| 3 T443929 | M | T3N0M0 | II | 70 | WT | MSS |
| 4 T449960 | M | T3N0M0 | II | 70 | G12S; GGT→AGT | MSS |
| 5 T449986 | M | T4N0M1 | IV | 50 | G12D; GGT→GAT | MSS |
| 6 T450178 | M | T3N2M0 | III | 60 | G13D; GGC→GAC | MSS |
| 7 T451238 | M | T4N1M1 | IV | 60 | G12D; GGT→GAT | MSS |
| 8 T456025 | M | T4N1M1 | IV | 60 | G12D; GGT→GAT | MSS |
| 9 T458032 | M | T4N1M0 | III | 60 | G12D; GGT→GAT | MSS |
| 10 T455203 | F | T3N0M0 | II | 80 | WT | MSI |
| 11 T454071 | F | T3N0M0 | II | 70 | G12V; GGT→GTT | MSS |
| 12 T455333 | F | T3N0M0 | II | 60 | G12A; GGT→GCT | MSS |
| 13 T449864 | M | T2N0M0 | I | 70 | WT | MSS |
| 14 T454871 | M | T3N2M0 | III | 50 | G12S; GGT→AGT | MSS |
| 15 T452699 | M | T2N1M0 | III | 90 | G13D; GGC→GAC | MSS |

**Additional_file_1 as DOC**
**Additional file 1** Table S1
