# Supplementary material for: Aberrant methylation of NPY, PENK, and WIF1 as a promising marker for blood-based diagnosis of colorectal cancer
Source: BMC Cancer. 2013 Dec 1;13:566. doi: 10.1186/1471-2407-13-566 (PMC4219483; doi:10.1186/1471-2407-13-566)
Supplement: Additional file 2: Table S2 — Oligonucleotides. [file 1471-2407-13-566-S2.doc]

**Additional_file_2 as DOC**
**Additional file 2** Table S2
