# Supplementary material for: Aberrant methylation of NPY, PENK, and WIF1 as a promising marker for blood-based diagnosis of colorectal cancer
Source: BMC Cancer. 2013 Dec 1;13:566. doi: 10.1186/1471-2407-13-566 (PMC4219483; doi:10.1186/1471-2407-13-566)
Supplement: Additional file 3: Figure S1 — Selection of candidate biomarkers by DNA methylation-array. Left: Venn diagram. Urine, Serum and Tissue lists obtained by taking the top decile in the ranked Ca-N lists. Right: Loci Illumina goldengate IDs. [file 1471-2407-13-566-S3.doc]

**Additional_file_3 as DOC**
**Additional file 3** Figure S1
