# Supplementary material for: Aberrant methylation of NPY, PENK, and WIF1 as a promising marker for blood-based diagnosis of colorectal cancer
Source: BMC Cancer. 2013 Dec 1;13:566. doi: 10.1186/1471-2407-13-566 (PMC4219483; doi:10.1186/1471-2407-13-566)
Supplement: Additional file 4: Figure S2 — The bisulfite sequence of the WIF1 promoter. Representative bisulfite sequencing electrophoregram of the WIF1 promoter verifies methylation status assessed by QS-MSP from carcinoma colorectal (Tumor) and adjacent normal tissues (Adj Norm). The diagram above illustrates one of the 15 samples of tumor tissues; cytosine nucleotides underlined in black remain unchanged indicating all sites are methylated in the amplicon product. By contrast, in the homologous normal tissue, only thymidine nucleotides underlined in red are detected instead of cytosine residues due to bisulfite modified DNA which is indicative of unmethylated amplicon products. It is interesting to note that comparison of two sequences of normal and tumoral tissues indicates that all cytosine at non-CpG sites are converted to thymine resulting entirely from DNA modification. This follows after sodium bisulfite treatment when referring to the wild-type WIF1 gene sequence. [file 1471-2407-13-566-S4.doc]

**Additional_file_4 as DOC**
**Additional file 4** Figure S2
