# Supplementary material for: Aberrant methylation of NPY, PENK, and WIF1 as a promising marker for blood-based diagnosis of colorectal cancer
Source: BMC Cancer. 2013 Dec 1;13:566. doi: 10.1186/1471-2407-13-566 (PMC4219483; doi:10.1186/1471-2407-13-566)
Supplement: Additional file 5: Table S3 — Gene promoter analysis in adjacent normal and tumor tissues. Optimal threshold values obtained for simple gene and in combination of 3 genes. Taking into account the different degrees of methylation, we set threshold of CMI at 58% to obtain the highest performance in terms of sensitivity and specificity. [file 1471-2407-13-566-S5.doc]

**Additional_file_5 as DOC**
**Additional file 5** Table S3
