# Supplementary material for: Aberrant methylation of NPY, PENK, and WIF1 as a promising marker for blood-based diagnosis of colorectal cancer
Source: BMC Cancer. 2013 Dec 1;13:566. doi: 10.1186/1471-2407-13-566 (PMC4219483; doi:10.1186/1471-2407-13-566)
Supplement: Additional file 6: Figure S3 — Methylation correlated in stages of CRC. Mean cumulative methylation in I / II and III / IV stages of CRC serum samples. Differences between both stages were not significant (P > 0.1, Student-test). Plotted is the mean (± SD; bars) amount of cumulative methylation in I / II stages with mean = 44.40 ± 78.53, versus in III / IV stages with mean = 33.55 ± 61.71. [file 1471-2407-13-566-S6.doc]

**Additional_file_6 as DOC**
**Additional file 6** Figure S3
